# Institutional Housekeeping

**Published:** 1911-12-16

**Authors:** 


### INSTITUTIONAL HOUSEKEEPING.

(Contributions invited: Workers' Questions will be welcome.)

### How to Avoid Wasting Fat.

Unless there is careful supervision, fat is either very much wasted or it mysteriously disappears. Whenever large joints of meat are cooked there must be dripping, though careful cooking and basting can minimise the amount drawn from the meat. It is an important part of the housekeeper's or steward's duty to take continual precautions against the butcher sending an undue proportion of fat with the meat. Any superfluous fat should be trimmed off before cooking, and with boiled meats very little fat should be left. Invalids especially ought not to have rich fat served with their diets. During roasting, care must be taken that the fat which comes from the meat does not burn. A double baking-tin with water in the under tin will obviate this. Meat cooked in a gas oven has a special dripping tin placed under it, away from the great heat. After the meat is cooked, the liquid fat should be poured into a basin containing water; the particles sink, and the fat when cool is a firm white cake; this must be removed from the water and scraped underneath. It can then be put aside for use.

All trimmings of fat from joints or cutlets left over in the kitchen, after carving from cooked or uncooked meat, should be cut up into pieces about an inch square, all red or discoloured pieces being sorted out together with any scraps of meat, and the squares of pure fat should then be placed in a strong iron cooking-pot and covered with water. The water should be allowed to boil, and must be constantly skimmed to free it from all impurities. After a time the water boils away and the pieces of fat begin to look shrivelled and turn golden-brown. There is a thick-looking liquid in the pan and care must be taken in stirring as the fat is apt to splutter at this stage. The fat is allowed to cool slightly and is then strained off into a basin. Even the pieces in the strainer may be utilised, as when cold they make an excellent substitute for suet, and when chopped finely can be used for making such puddings as baked raisin or baked bread, which are naturally rather dark in colour.

Six pounds of fat take about five hours' slow cooking. When strained and cooled it forms a firm white cake known as clarified fat, and can take the place of butter for many purposes. It needs a little care in preparation, for it must on no account be allowed to burn; it must be stirred occasionally, and if it begins to throw up a faint blue smoke during the cooking process the heat must be reduced. In summer weather, to prevent the dripping from roast meat from decomposing when kept, it should be turned into a pan, with boiling water added, and should then be well stirred and boiled. When cool the cake of fat should be taken off and scraped underneath.

Dark-coloured dripping, or such as is full of particles, should be clarified with the other pieces of fat. Cooked fat takes less time to clarify, so that if a large quantity of raw fat were being clarified it would be better to add the cooked fat later.

Clarified fat or dripping can be utilised in many ways. Spread on bread or toast instead of butter it is popular with children. It makes delicious light cakes, buns, and scones. Pastry is also light and short when made from it, in the proportion of half a pound of fat to one pound of flour. Many light puddings can also be made with dripping. If melted and beaten up well before being added to cakes, or shredded finely before being made into pastry, the taste of fat will hardly be noticed; and if carefully clarified it is a good colour, so that it does not spoil the appearance of any mixture. It is, however, not so suitable for boiled puddings, and for this reason cannot as a rule take the place of suet.

For frying purposes it is most useful. It does not burn so quickly as butter, and it renders the food less greasy than lard, nor does it impart a fatty flavour. For deep frying sufficient fat should be placed in a strong iron utensil completely to cover whatever is to be fried. It must heat slowly until a faint smoke rises, and then the food is immersed in it. This is a very satisfactory method for heating rissoles, fish, or food requiring little cooking. It is not wasteful, for if strained after use, the fat, if not burned, may be used again. Melted fat, water, and flour form an inexpensive batter for coating fish before frying in deep fat.

The fat used for sweet mixtures such as cakes or fruit tarts must have no distinctive flavour, and hence the dripping from stuffed meats or bacon fat can only be utilised for savoury preparations. The neglect of this precaution is responsible for much prejudice against the use of dripping.

Each day the housekeeper should note what dripping and fat are left over and utilise it accordingly. It is most difficult to prevent fat being treated as a perquisite, and it is very necessary on engaging a cook to make her fully understand that all the fat and dripping are to be used in the household.

---

"Holbein."—It is certainly cheaper to make your own furniture polish if you can spare time to do so. The following is one of the best and most economical preparations for the purpose which can be made at home. Into a two-quart bottle put a pint each of linseed oil and turpentine, and half a pint each of methylated spirit and brown vinegar. Shake all these well together until completely emulsified, and invariably shake the bottle before the polish is used. Such a polish, if bought, would cost about 1s. 6d. per half-pint, whereas made at home the price works out at about 1s. 6d. for three pints.

"Duffer" is in trouble about her new cork carpet, which shows every mark. The material being porous absorbs, like a sponge, every preparation which has been tried with the object of lending it a polish. We are sorry that we can only recommend patience. Constant wear will render the surface smoother after a time, and the dust will then cling less. Avoid the use of either paraffin or milk, as the former makes the room unpleasant for a long time, and the latter turns sour. Have the room constantly swept with a non-fluffy cloth, dipped in warm, slightly soapy water. But perhaps one of our readers can help "Duffer."

